# Supplementary figures and images for: Fibroblast Growth Factor Receptor Inhibitors Decrease Proliferation of Melanoma Cell Lines and Their Activity Is Modulated by Vitamin D
Source: Int J Mol Sci. 2024 Feb 21;25(5):2505. doi: 10.3390/ijms25052505 (PMC10931346; doi:10.3390/ijms25052505)

**A**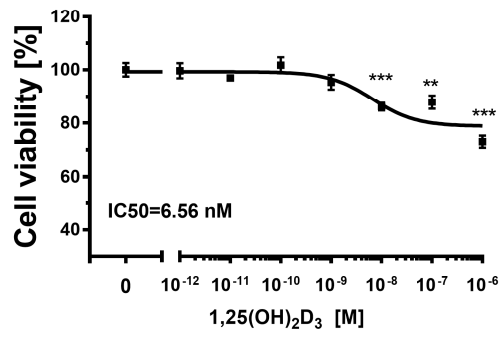**B**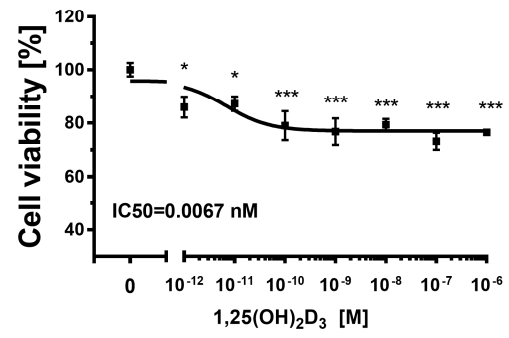

**Figure S1.** Effect of 1,25(OH)<sub>2</sub>D<sub>3</sub> on viability of RPMI7951 melanoma. \*  $p < 0.05$ , \*\*  $p < 0.01$ , \*\*\*  $p < 0.001$ .

Supplement: Supplementary file 1 [file ijms-25-02505-s001.zip › ijms-2866566-supplementary.pdf]
